# Development of a prognostic nomogram for esophageal squamous cell carcinoma patients received radiotherapy based on clinical risk factors

**DOI:** 10.3389/fonc.2024.1429790

**Published:** 2024-08-22

**Authors:** Yang Li, Xian Shao, Li-Juan Dai, Meng Yu, Meng-Di Cong, Jun-Yi Sun, Shuo Pan, Gao-Feng Shi, An-Du Zhang, Hui Liu

**Affiliations:** ^1^ Department of Computed Tomography and Magnetic Resonance Imaging, The Fourth Hospital of Hebei Medical University, Hebei, Shijiazhuang, China; ^2^ Department of Anesthesiology, The Fourth Hospital of Shijiazhuang, Hebei, Shijiazhuang, China; ^3^ The Second Hospital of Hebei Medical University, Shijiazhuang, Hebei, China; ^4^ Department of Computed Tomography and Magnetic Resonance Imaging, Hebei Children’s Hospital, Shijiazhuang, Hebei, China; ^5^ Department of Radiology, First Hospital of Qinhuangdao, Hebei, Qinhuangdao, China; ^6^ Department of Radiotherapy, The Fourth Hospital of Hebei Medical University, Hebei, Shijiazhuang, China

**Keywords:** carcinoma, squamous cell, esophageal neoplasms, radiotherapy, locoregional recurrence-free survival, nomogram

## Abstract

**Purpose:**

The goal of the study was to create a nomogram based on clinical risk factors to forecast the rate of locoregional recurrence-free survival (LRFS) in patients with esophageal squamous cell carcinoma (ESCC) who underwent radiotherapy (RT).

**Methods:**

In this study, 574 ESCC patients were selected as participants. Following radiotherapy, subjects were divided into training and validation groups at a 7:3 ratio. The nomogram was established in the training group using Cox regression. Performance validation was conducted in the validation group, assessing predictability through the C-index and AUC curve, calibration via the Hosmer-Lemeshow (H-L) test, and evaluating clinical applicability using decision curve analysis (DCA).

**Results:**

T stage, N stage, gross tumor volume (GTV) dose, location, maximal wall thickness (MWT) after RT, node size (NS) after RT, Δ computer tomography (CT) value, and chemotherapy were found to be independent risk factors that impacted LRFS by multivariate cox analysis, and the findings could be utilized to create a nomogram and forecast LRFS. the area under the receiver operating characteristic (AUC) curve and C-index show that for training and validation groups, the prediction result of LRFS using nomogram was more accurate than that of TNM. The LRFS in both groups was consistent with the nomogram according to the H-L test. The DCA curve demonstrated that the nomogram had a good prediction effect both in the groups for training and validation. The nomogram was used to assign ESCC patients to three risk levels: low, medium, or high. There were substantial variations in LRFS between risk categories in both the training and validation groups (p<0.001, p=0.003).

**Conclusions:**

For ESCC patients who received radiotherapy, the nomogram based on clinical risk factors could reliably predict the LRFS.

## 1 Introduction

Esophageal cancer (EC) is a frequent illness that originates in the upper digestive system and is a type of malignant tumor that is rather prevalent. It has an extremely high incidence rate and fatality rate (1, 2). The staging system was very important for clinical treatment and prognosis. Even if the treatment plan was similar, there are some differences in TNM staging, which will have a certain impact on the predictive value (3), which might be related to tumor heterogeneity and radiotherapy sensitivity. It was thus necessary to construct a more precise and practical predictive model for EC patients receiving radical radiotherapy (RT) in order to better personalize treatment options based on the risk of locoregional recurrence or mortality. To the best of our knowledge, the vast majority of studies were based on pretreatment features (4) and did not take into account after-treatment features. The tumor status after RT was usually related to the therapeutic effect (5, 6). Therefore, when establishing the prediction model, the symptoms or tumor response after treatment could be used as an indicator, and stages could study the symptoms of esophageal squamous cell carcinoma (ESCC) patients, and the corresponding treatment plan could be provided. A nomogram was regarded as an accurate instrument for measuring individual risk (7). It has previously been demonstrated to exhibit accurate prediction capacity and has been used in practice to determine the prognosis of a variety of kinds of cancer (7–10). Therefore, based on clinical risk factors and computer tomography (CT) characteristics, the current study constructed a nomogram to predict locoregional recurrence-free survival (LRFS) in ESCC patients who underwent radical (chemo)radiotherapy. Independent internal cohorts were also employed in the study to validate the final model.

## 2 Materials and methods

### 2.1 Inclusion criteria and study population

**Figure 1:**
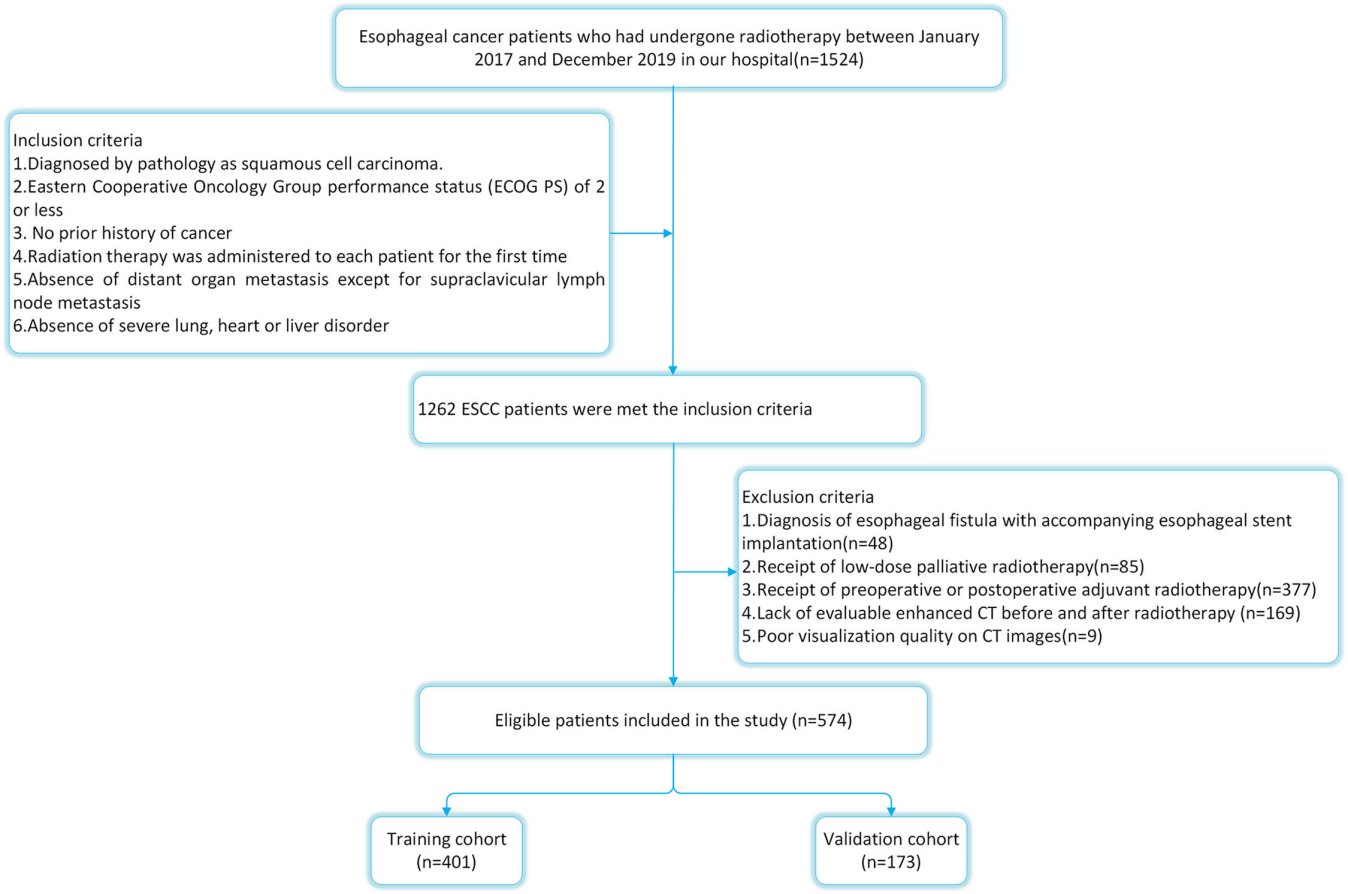
The flowchart of the patients enrolled in our study.

The ethical approval for this study was obtained from the ethics committee of the Fourth Hospital of Hebei Medical University. Because ESCC accounted for almost 90% of all EC instances in China (11), we limited our analysis to patients with ESCC. From January 2017 to December 2019, 574 ESCC patients who underwent radical (chemo)radiotherapy at the Fourth Hospital of Hebei Medical University were chosen as participants. The selection criteria were as follows: 1) all subjects have been diagnosed by pathology; 2) the Eastern Cooperative Oncology Group (ECOG) score shall not exceed 2; 3) no prior history of cancer or other illnesses that might have impacted treatment; 4) routine imaging tests revealed no distant organ metastases (MRI for brain, CT for lung and liver, and bone scan; 5) radiation therapy was administered to each patient for the first time; 6) metastasis of supraclavicular lymph nodes was not excluded. The exclusion criteria were as follows:1) diagnosis of esophageal fistula with accompanying esophageal stent implantation (n=48); 2) receipt of low-dose palliative radiotherapy (n=85); 3) receipt of preoperative or postoperative adjuvant radiotherapy(n=377); 4) lack of evaluable enhanced CT before and after radiotherapy (n=169); 5) poor visualization quality on CT images(n=9). The detailed flow chart of patient selection is shown in **Figure 1**.

### 2.2 Treatment

RT alone was administered to patients who were too old, had poor performance, or requested it. 374 patients among the subjects accepted the chemoradiotherapy (CRT) treatment scheme. The protocols should be followed with regard to dose restrictions for the organs at risk and the definition of the radiation target volume. Three-dimensional conformal or intensity-modulated radiotherapy was used to carry out all of the treatment plans. The total group’s planning target volume (PTV) and gross tumor volume (GTV) prescription radiation doses ranged from 50.0 to 66.0 Gy (median 60 Gy). PTV was given 1.8-2.0 Gy/fraction, while GTV was given 1.95-2.15 Gy/fraction, 5 times per week. The physiotherapist completed the treatment plan as needed, and a senior physician approved it. The following was the specific treatment scheme (12): cisplatin combined with 5-fluorouracil, cisplatin combined with paclitaxel and TS-1. The final choice of chemotherapy treatment plan was mainly due to the results of expert decision and their own treatment intention.

### 2.3 CT Image acquisition and evaluation

The contrast-enhanced CT scans were performed within one month before and after radiotherapy. Two commercial CT scanners were used to gather all the CT images. Scanner 1: a single-tube, dual-source CT of the second generation (SOMATOM Definition Flash, Siemens Healthcare, Forchheim, Germany). The subsequent scanning protocols were employed: tube voltage of 120 kVp, automatic mA, slice thickness of 5.0 mm, increments of 5.0 mm, rotational speed of 0.5 seconds, pitch of 1.2 mm, reconstruction algorithm of b20-40f, and thickness of reconstruction section of 1-1.25 mm. Scanner 2: 256-slice CT scanner, using the common single-energy mode (Revolution CT, GE Healthcare, Milwaukee, USA). The following were the scanning parameters: tube voltage of 120 kVp, automated mA, slice thickness of 5.0 mm, increment of 5.0 mm, rotation time of 0.5 seconds, pitch of 0.992:1, standard reconstruction method, and 1.25 mm reconstruction section thickness. All patients were lying supine throughout the scan, which covered the chest or the chest and abdomen. 30 seconds after intravenous injection of contrast agent (3.0-4.0 ml/s, 1.5 ml/kg, Iohexol, 300 mg I/mL), an arterial phase scan was performed using a syringe pump, followed by an injection of 20 ml of saline for washout.

**Figure 2:**
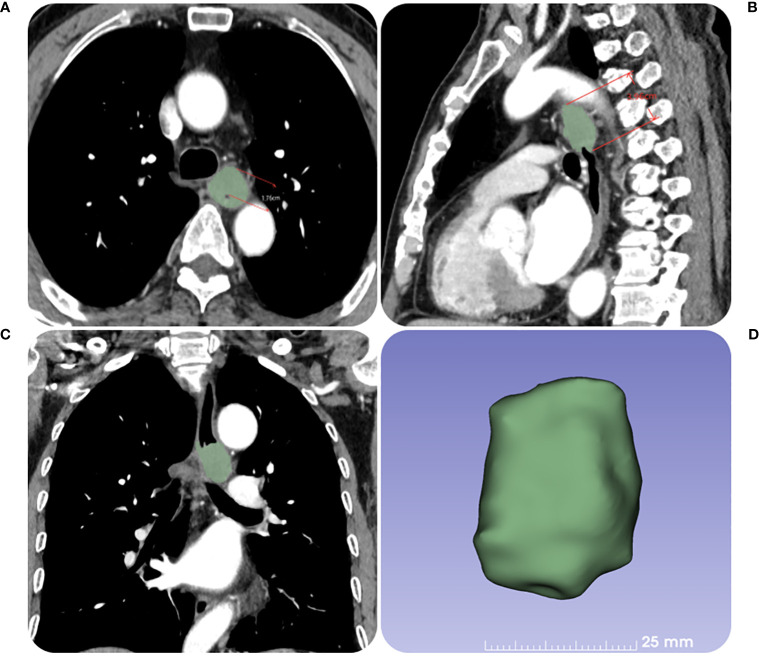
The contrast-enhanced CT images of a 67-year-old female patient with ESCC before RT. **(A)** The tumor maximal wall thickness is measured in the transverse axial position. **(B, C)** The tumor length is measured in the sagittal position with reference to the coronal position. **(D)** The tumor volume is obtained by outlining the tumor layer by layer.

**Figure 3:**
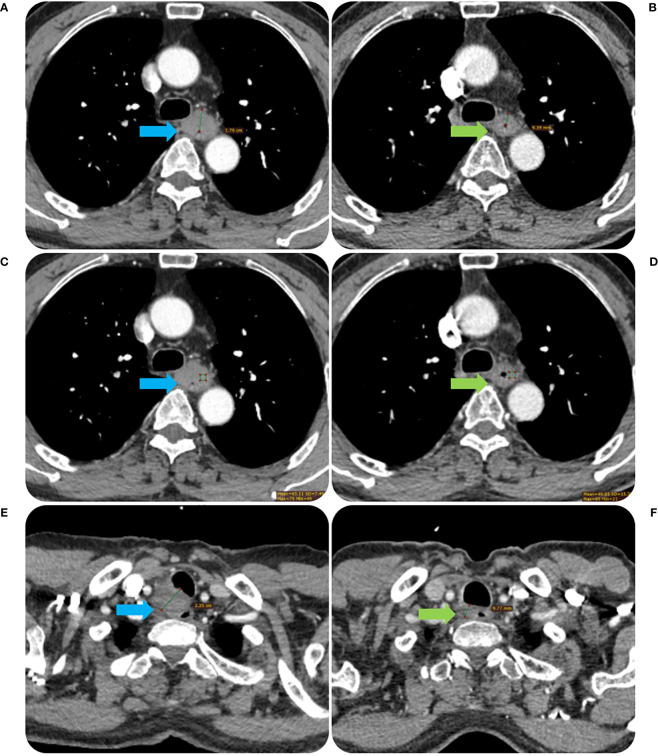
The contrast-enhanced CT images before and after RT of the same patient in **Figure 2**. The tumor MWT was 1.76 cm before RT treatment **(A)** and decreased to 0.94 cm after treatment **(B)**. The CT value of the tumor was 65.11 Hu before RT treatment **(C)** and decreased to 48.85 Hu after treatment **(D)**. The short diameter of the metastatic lymph node in the right proper supra tracheoesophageal sulcus was about 2.25 cm before RT treatment **(E)**, which was reduced to 0.98 cm after treatment **(F)**.

The Radiant DICOM viewer (free software; available online at https://www.radiantviewer.com) was used to upload the thin-slice CECT pictures. The entire tumor was displayed to the greatest extent possible in the sagittal plane using the multi-planar reconstruction mode. The short axis of the biggest lymph node observed in CT images was used to calculate node size, and tumor length was computed based on the axis image. The maximal esophageal wall thickness of the tumor and the short axis of the node at the same level in the area in the transverse section were measured in accordance with the position of the tumor and lymph node before RT. In the transverse slice, a credible tumor region of interest (ROI) was identified within the representative thickened esophageal wall. The software on this image processing workstation automatically calculated the tumor attenuation value. The ROIs covered the largest possible portion of the most noticeably increased area. The tumor’s ulceration and necrosis, gas in the lumen of the tumor, blood vessels, and the adipose around the lesions were all avoided as much as possible when drawing the ROIs. Three sequential tiers of measurements were made. ΔCT value was defined as the relative change of tumor attenuation value before and after RT. All the CT images were analyzed by a radiologist and radiotherapist (with 11 and 12 years of experience, respectively), and when their assessments disagreed, agreement was reached after consultation. The detailed measurement process is shown in **Figures 2**, **3**.

### 2.4 Follow-up

The final result of the study was LRFS. The time between the start of radiation and the first local and/or regional failure, the last follow-up, or the date of death from any cause was referred to as the LRFS. The subsequent examination mainly included routine blood tests, imaging examinations, and barium swallows. A chest-abdomen CT was used to diagnose recurrence or lymph node metastasis, and a tissue biopsy of suspected recurrent lesions was performed if clinically possible.

### 2.5 Design and statistical analysis

According to the 7:3 ratio (13–15), patients with ESCC were divided into groups for training and validation. Mann-Whitney U-test or T-test was used to assess the continuous variables, and Fisher’s exact test or χ2 test was used to compare the training cohort with the validation cohort.

The Cox multivariate analyses were employed to construct the nomogram within the training cohort and subsequently validate its performance in the validation group. Independent prognostic indicators for LRFS were determined using hazard ratios (HRs) derived from a Cox proportional hazards regression model.

The bootstrap method was utilized with 1000 resamples for calculations, generating a calibration graph to assess the expected performance characteristics of the nomogram.

The assessment of the model’s discriminatory ability between the nomogram and conventional TNM staging was done through Harrell’s consistency index (C-index) and the area under the receiver operating characteristic (AUC) curve. Delong test was employed to compare the AUCs between the models. To assess the fit of the model, the Hosmer-Lemeshow goodness-of-fit test was performed. The nomogram’s clinical validity was established using decision curve analysis (DCA), which determined net benefits across various threshold probabilities.

Based on the nomogram’s ratings for each important variable, the overall score is determined. X-Tile software (version 3.6.1) identified the optimal cutoff value for the survival risk score, and three categories were created using the LRFS risk ratings (low-risk, middle-risk and high-risk). The Kaplan-Meier estimator was used to estimate LRFS, and the log-rank test was used to compare them. The significance level for each two-sided test was set at p<0.05. R version 4.2.1 software was utilized to execute any statistical calculations (The R Foundation for Statistical Computing, Vienna, Austria).

## 3 Results

### 3.1 Characteristics of the patient

This study included 574 patients in total. All patients had been followed for more than two years as of May 1, 2022, with a median follow-up of 45 months. The overall rate of follow-up was 96.69%. **Table 1** shows the characteristics of ESCC patients in the training and validation groups, with no data differences.

**Table 1:** Characteristics of patients in training cohort and validation cohort.

Characteristics	Training cohort (n=401)	Validation cohort (n=173)	P
ECOG			0.751*
0-1	249(62.1%)	105(60.7%)	
2	152(37.9%)	68(39.3%)	
Age			0.430*
≤65	146(36.4%)	69(39.9%)	
>65	255(63.6%)	104(60.1%)	
Sex			0.822*
Male	251(62.6%)	110(63.6%)	
Female	150(37.4%)	63(36.4%)	
Smoking			0.706*
Yes	183(45.6%)	76(43.9%)	
No	218(54.4%)	97(56.1%)	
Alcohol			0.972*
Yes	157(39.2%)	68(39.3%)	
No	244(60.8%)	105(60.7%)	
Family history			0.969*
Yes	91(22.7%)	39(22.5%)	
No	310(77.3%)	134(77.5%)	
Location			0.612*
Cervical	17(4.2%)	10(5.8%)	
Upper	140(34.9%)	58(33.5%)	
Middle	196(48.9%)	79(45.7%)	
Lower	48(12.0%)	26(15.0%)	
T Stage			0.954*
T2	47(11.7%)	19(11.0%)	
T3	189(47.1%)	81(46.8%)	
T4a	78(19.5%)	37(21.4%)	
T4b	87(21.7%)	36(20.8%)	
N Stage			0.450*
N0	18(4.5%)	10(5.8%)	
N1	82(20.4%)	42(24.3%)	
N2	202(50.4%)	75(43.4%)	
N3	99(24.7%)	46(26.6%)	
Supraclavicular LN			0.410*
Yes	63(15.7%)	32(18.5%)	
No	338(84.3%)	141(81.5%)	
TNM			0.786*
II	32(8.0%)	12(6.9%)	
III	128(31.9%)	53(30.6%)	
IVA	180(44.9%)	76(43.9%)	
IVB	61(15.2%)	32(18.5%)	
Tumor length (cm)	7.59 ± 2.19	7.71 ± 2.13	0.437 #
Tumor volume (cm^3^)	77.27 ± 56.02	78.11 ± 61.32	0.963 #
MWT before RT (cm)	2.00 ± 0.57	1.99 ± 0.61	0.581 #
MWT after RT (cm)	1.48 ± 0.42	1.49 ± 0.46	0.401 #
NS before RT (cm)	1.23 ± 0.66	1.25 ± 0.76	0.704 #
NS after RT (cm)	0.96 ± 0.49	0.97 ± 0.64	0.292 #
CT Value before RT(HU)	70.45 ± 13.25	70.04 ± 12.62	0.604 #
CT Value after RT(HU)	57.90 ± 15.56	58.02 ± 15.18	0.707 #
ΔCT value			0.209*
≤0.35	318(79.3%)	145(83.8%)	
>0.35	83(20.7%)	28(16.2%)	
Chemotherapy			0.958*
Yes	261(65.1%)	113(65.3%)	
No	140(34.9%)	60(34.7%)	
GTV dose (Gy)			0.078*
50-60	92(22.9%)	30(17.3%)	
60	189(47.1%)	99(57.2%)	
60-66	120(29.9%)	44(25.4%)	
PTV dose (Gy)			0.564*
50-60	169(42.1%)	76(43.9%)	
60	167(41.6%)	75(43.4%)	
60-66	65(16.2%)	22(12.7%)	

*, χ2 test; #, Mann-Whitney U-test.

### 3.2 Cox regression analyses

**Figure 4:**
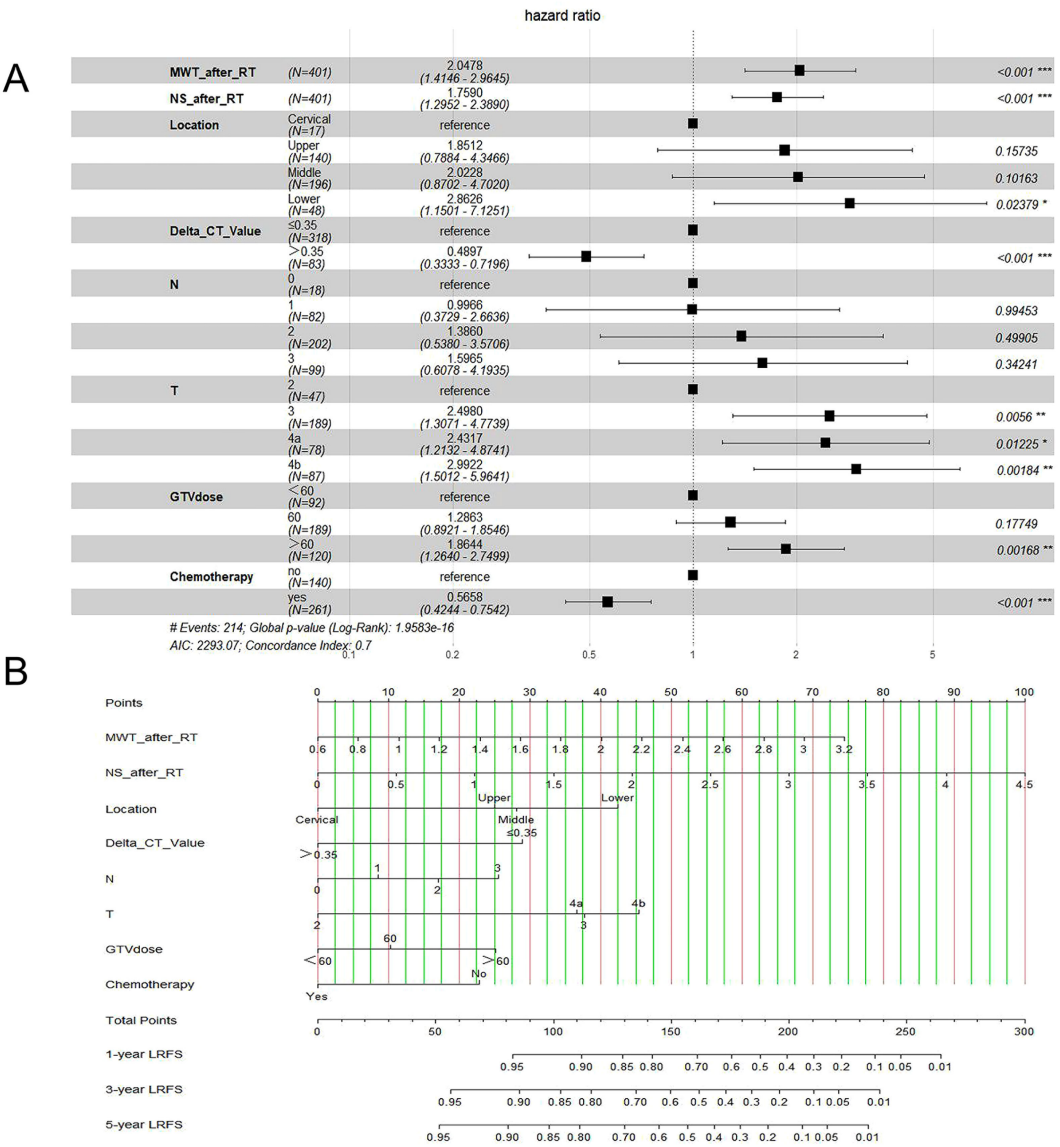
Forest plot of Cox multivariate analyses **(A)**; Nomogram for LRFS **(B)**.

In this study, risk factors included parameters as below: ECOG, age, sex, history of alcohol and tobacco, family history, tumor length (TL), location of the tumor, and tumor volume (TV), T stage, N stage, supraclavicular lymph node, TNM stage, PTV dose, GTV dose, whether received chemotherapy, maximal wall thickness (MWT) before RT, MWT after RT, node size (NS) before RT, NS after RT, CT value before RT, CT value after RT, ΔCT value. **Table 2** shows LRFS factor-specific univariate and multivariate Cox regression models. Alcohol history, tumor location, TL, TV, T stage, N stage, TNM stage, GTV dose, if chemotherapy was given, MWT before RT, MWT after RT, NS before RT, NS after RT, CT value before RT, and ΔCT value were predictive indicators for LRFS in the training group’s univariable analysis (p<0.05). The results of the multivariable analysis revealed that the following factors could independently predict LRFS: tumor location (P=0.046), T stage (P=0.049), N stage (P=0.038), GTV dose (P=0.003), MWT after RT (P=0.002), NS after RT (P=0.036), ΔCT value (P=0.05), and whether patients received chemotherapy (P<0.001) (**Figure 4A**).

**Table 2:** Univariate and multivariate Cox regression for LRFS in patients.

Characteristics	Univariate analysis			Multivariate analysis		
	HR	95%CI	P	HR	95%CI	P
WT before RT	1.69	1.37 - 2.09	<0.001	0.68	0.25 - 1.88	0.459
MWT after RT	2.86	2.09 - 3.93	<0.001	2	1.29 - 3.1	0.002
NS before RT	1.52	1.29 - 1.8	<0.001	1.12	0.81 - 1.54	0.493
NS after RT	2.08	1.61 - 2.69	<0.001	1.61	1.03 - 2.52	0.036
TL	1.15	1.08 - 1.22	<0.001	0.99	0.86 - 1.14	0.936
TV	1.01	1 - 1.01	<0.001	1.01	0.99 - 1.02	0.418
CT Value before RT	0.98	0.97 - 0.99	0.001	0.99	0.98 - 1.01	0.342
Alcohol			0.049			0.390
No	Ref.			Ref.		
Yes	1.31	1 - 1.72		0.87	0.64 - 1.19	
ΔCT value			0.014			0.065
≤0.35	Ref.			Ref.		
>0.35	0.63	0.43 - 0.91		0.55	0.3 - 1.04	
Location			0.018			0.046
Cervical	Ref.			Ref.		
Upper	1.515	0.658-3.489		1.769	0.74-4.21	
Middle	1.811	0.796-4.121		1.805	0.76-4.27	
Lower	2.284	0.948-5.505		2.441	0.96-6.19	
T stage			<0.001			0.049
2	Ref.			Ref.		
3	3.224	1.731-6.007		2.868	1.29-6.39	
4a	2.982	1.532-5.805		2.746	1.09-6.92	
4b	4.559	2.393-8.687		3.156	1.27-7.84	
N stage			0.001			0.038
0	Ref.			Ref.		
1	1.887	0.738-4.826		1.011	0.30-3.36	
2	2.952	1.205-7.229		1.349	0.39-4.63	
3	3.589	1.439-8.951		1.554	0.44-5.51	
TNM stage			<0.001			0.676
II	Ref.			Ref.		
III	2.687	1.289-5.598		0.997	0.33-3.03	
IVa	3.497	1.703-7.180		1.041	0.32-3.34	
IVb	3.387	1.567-7.322		0.969	0.30-3.13	
GTV dose			0.021			0.003
50-60	Ref.			Ref.		
60	1.078	0.754-1.541		1.282	0.88-1.86	
60-66	1.499	1.033-2.175		1.910	1.29-2.84	
Chemotherapy			0.007			<0.001
No	Ref.			Ref.		
Yes	0.68	0.52 - 0.9		0.58	0.43 - 0.77	

### 3.3 Development of a nomogram based on clinical risk factors for the prediction of LRFS

The findings revealed that the independent prognostic variables for ESCC included tumor site, T stage, N stage, GTV dose, MWT after RT, NS after RT, ΔCT value, and whether patients received chemotherapy. Therefore, by fitting these clinical factors, we created LRFS prediction models. A worse prognostic factor was reflected by a greater nomogram score (**Figure 4B**).

### 3.4 Nomogram prediction performance in training and validation cohorts

**Figure 5:**
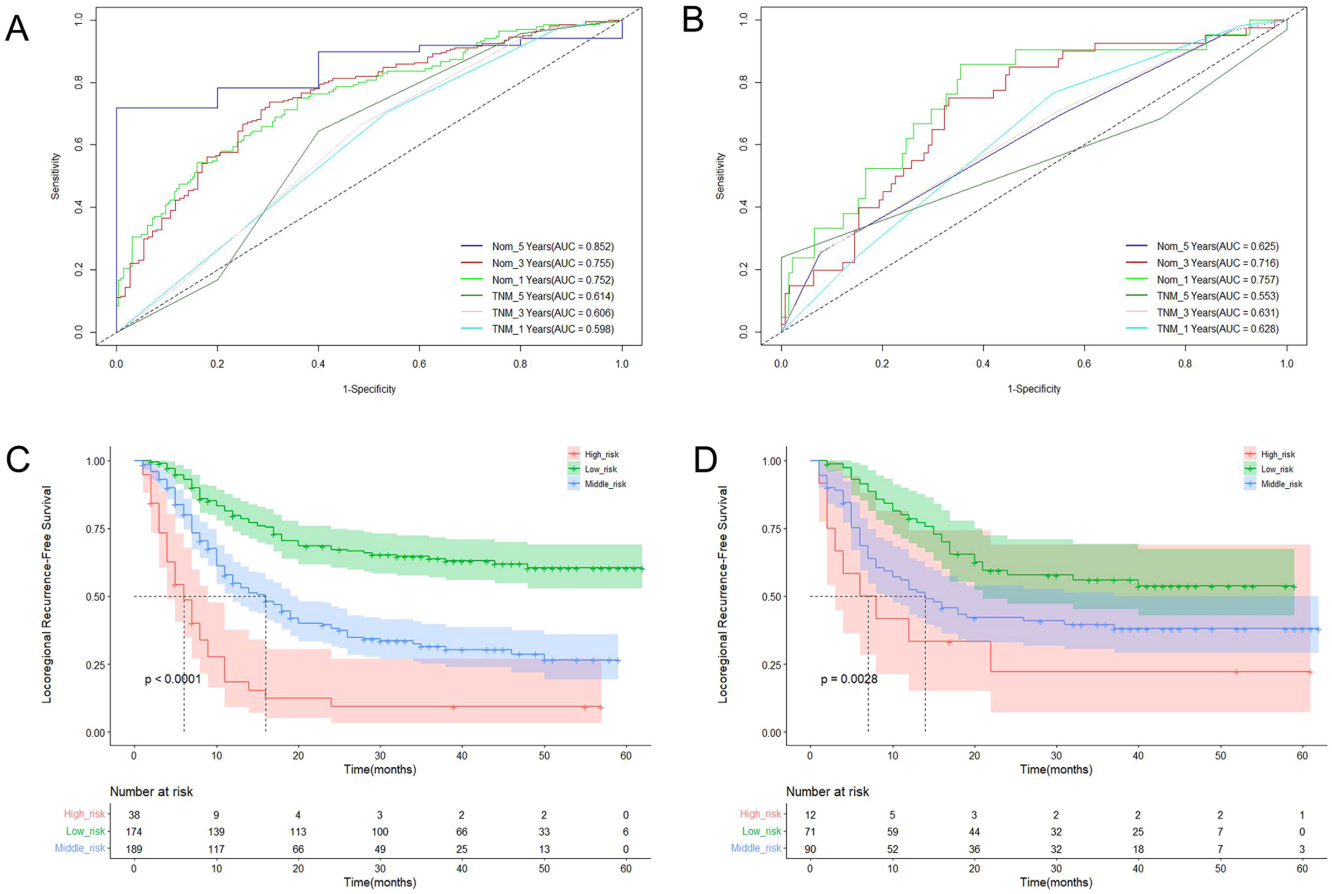
The ROC curves of LRFS based on the nomogram and TNM stage in training cohort **(A)** and validation cohort **(B)**; Kaplan-Meier curves for LRFS in training cohort **(C)** and validation cohort **(D)**.

**Figure 6:**
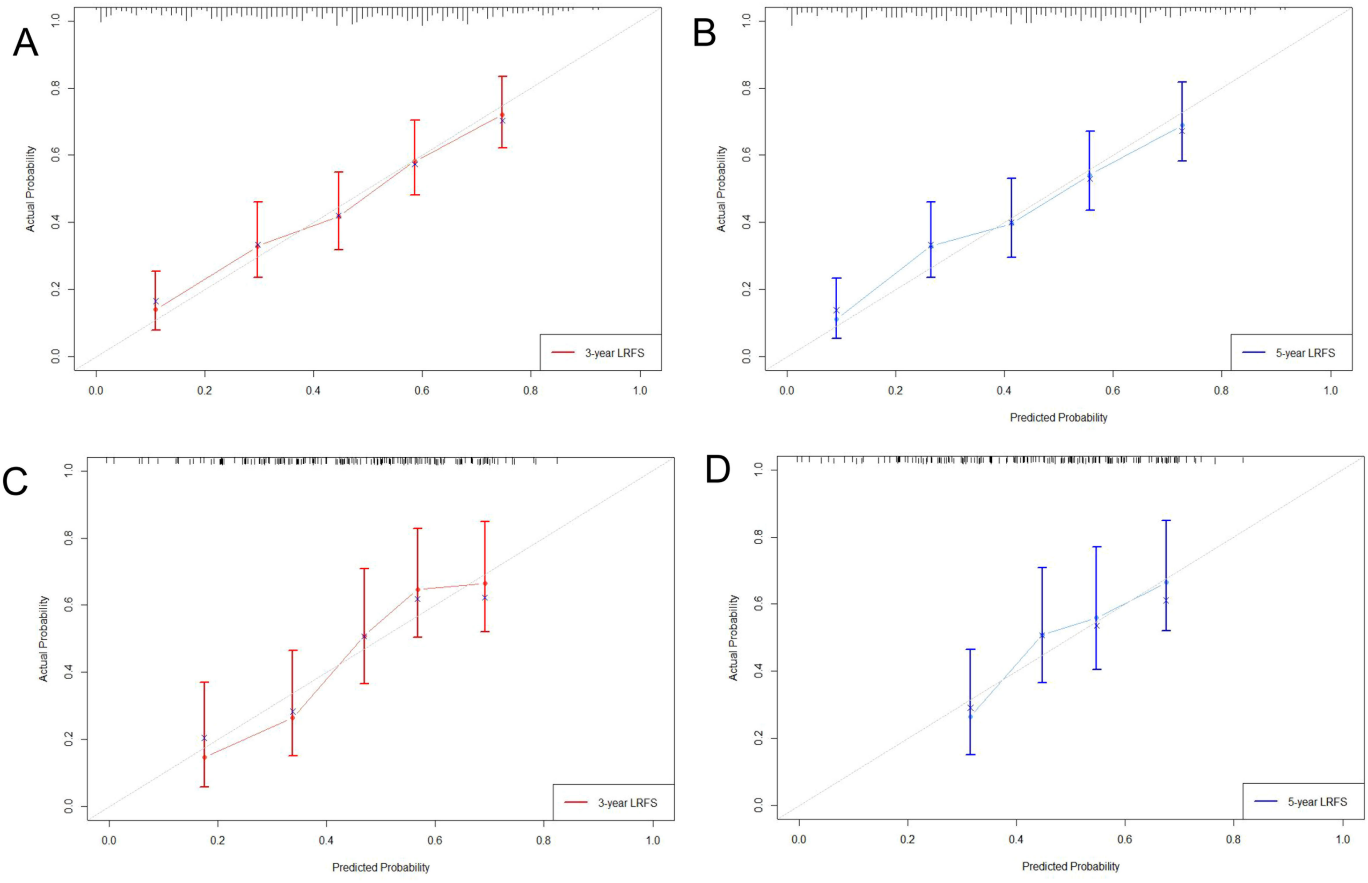
Calibration curve of 3-year **(A)**, 5-year **(B)** LRFS for training cohort; Calibration curve of 3-year **(C)**, 5-year **(D)** LRFS for validation cohort.

**Figure 7:**
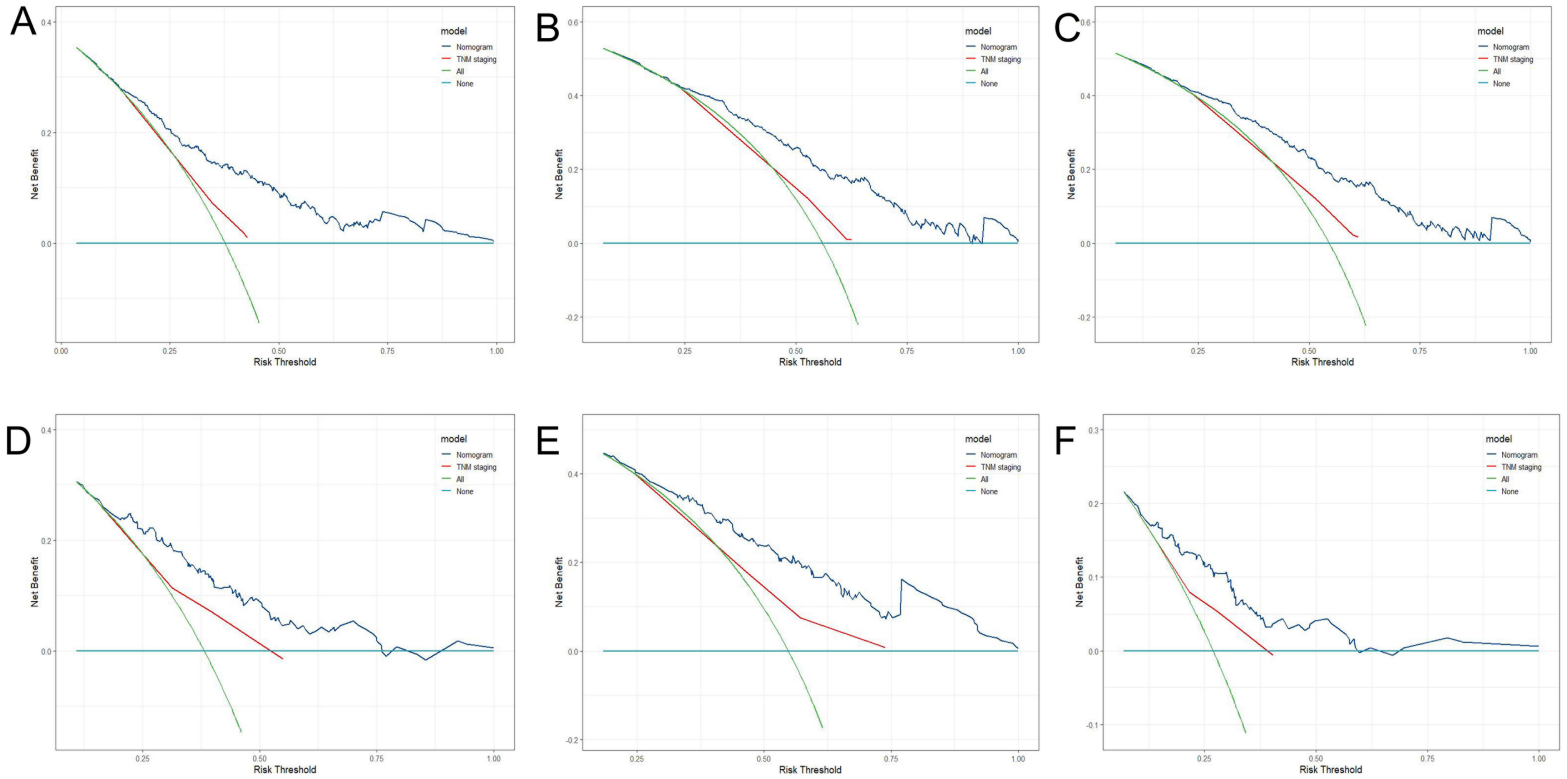
DCA of 1-year **(A)**, 3-year **(B)**, 5-year **(C)** LRFS for training cohort; DCA of 1-year **(D)**, 3-year **(E)**, 5-year **(F)** LRFS for validation cohort.

Three criteria were used to evaluate the nomogram model: discrimination, calibration, and clinical validity. The predictive precision of the model of the nomogram was contrasted with the 8th AJCC TNM staging criterion. In the prediction model for the training cohort, the C index of LRFS was 0.705 (95% CI, 0.668-0.742), and the C index of the 8th TNM staging standard was 0.572 (95% CI, 0.535-0.609). The nomogram’s C-index was significantly higher than the 8th TNM staging standard (p<0.001). The AUCs for the 1-year, 3-year, and 5-year LRFS prediction models were 0.752, 0.755, and 0.852, respectively, while the AUCs for the 8th TNM stage standard of the one-year, three-year, and five-year LRFS are 0.598, 0.606, and 0.614 (**Figure 5A**). When it came to predicting LRFS in 1, 3, and 5 years, the forecasting model outperformed the staging standard of the 8th TNM (Delong test, p<0.001, p<0.001, p=0.035). The Hosmer-Lemeshow (H-L) test result for the calibration curve for the nomogram’s prediction of the probability of 1-, 3-, and 5-year LRFS indicated good agreement between the observed LRFS risks and the nomogram’s predictions (χ2 = -306.66, p = 1) (**Figures 6A, B**). The data was subjected to DCA to determine the model’s clinical effectiveness (**Figures 7A–C**). Based on the nomogram created in this study, the decision curve revealed that the threshold probabilities for ESCC patients at 1, 3, and 5 years were, respectively, 21.51-51.93%, 37.01-75.28%, and 35.63-73.60%. These findings show that adopting this nomogram to forecast LRFS could boost accuracy in comparison to ignoring these parameters or employing a subpar approach.

The C-index of LRFS in the prediction model was 0.699 (95% CI, 0.646-0.752) in the validation cohort, and the C-index of the 8th TNM staging standard was 0.601 (95% CI, 0.548-0.654). The nomogram’s C- index was significantly higher than the 8th TNM staging standard (p=0.001). The AUC of the prediction model for 1-, 3-, and 5-year LRFS was 0.757, 0.716, and 0.676, respectively, and the AUC of the 8th TNM staging criteria for 1-, 3-, and 5-year LRFS was 0.628, 0.631, and 0.583 (**Figure 5B**). Throughout the whole follow-up period, Delong test consistently indicated that the prediction nomogram model was superior to the clinical TNM stage (p = 0.039, p = 0.042, p=0.049). Similarly, the calibration curves used to predict the validation cohort’s 3- and 5-year LRFS (**Figures 6C, D**) showed good concordance between the observations and the nomogram’s predictions (χ2 = 2.216, p = 0.974, H-L test). At 1, 3, and 5 years, DCA revealed that the threshold probability for ESCC patients ranged from 23.91 to 47.49%, 38.24 to 67.89%, and 15.54 to 32.85%, respectively (**Figures 7D–F**). The nomogram performed more accurately in terms of LRFS prediction for ESCC patients who underwent radical (chemo)radiotherapy than the conventional TNM staging approach.

### 3.5 Risk classification according to nomogram score

Using X-Tile software, the LRFS of ESCC patients was divided into danger phases based on the nomogram score. The training group was divided into three groups based on the LRFS score standard: low risk (score ≤ 120), medium risk (score 120-170), and high risk (score >170). At 1, 3, and 5 years, the LRFS for the three groups in training cohort were 79.6%, 63.8%, 60.4% and 54.8%, 31.5%, 26.5% and 18.5%, 9.2% and 9.2% respectively (χ2 = 89.02, p<0.001) (**Figure 5C**). The low risk (score ≤ 120), medium risk (score 120-170), and high risk (score >170) ESCC patients were represented in the validation cohort by 78.6%, 55.9%, 53.7% and 52.4%, 39.6%, 38.0%, and 33.3%, 22.2%, 22.2% of patients, respectively (χ2 = 11.749, p=0.003) (**Figure 5D**).

## 4 Discussion

RT was the primary and recommended course of treatment for ESCC patients who were either unwilling or unable to undergo surgery. Local recurrence or failure was the main cause of poor treatment effects after RT (12, 16). Currently, the accepted classification approach for determining a patient’s prognosis is the TNM 8th staging system (17, 18). However, the prediction results of ESCC patients treated with RT need to be more accurate. Nomograms have been used to assess patient outcomes and risk factors for many years. Several previous studies found that nomograms outperformed the conventional TNM staging approach to forecast the prognosis of patients with malignant tumors (19–21). The bulk of the predicted models did not incorporate the prognostic component of after-treatment characteristics, despite the fact that multiple prior research published nomogram prediction models for ESCC patients who had (chemo)radiotherapy (22–24). In view of the preceding, we created and verified a clinical signature-based nomogram model for predicting LRFS status in ESCC patients receiving RT, as well as several after-treatment characteristics. Our work was significant because it presented a specific approach for predicting LRFS in ESCC patients following RT.

The T stage and N stage prior to RT were crucial for determining the prognosis and formulating the treatment plan. In our study, the T stage and N stage served as a standalone predictive predictor for LRFS. The prognosis of EC was also significantly impacted by the thickness of the esophageal tumor (25). Previous research (26–28) found that both pre- and post-RT maximal esophageal wall thickness was a predictor of survival and pCR after treatment, particularly in patients with locally advanced ESCC. In contrast to measuring esophageal wall thickness, Aleksandra (29) found that measuring post-contrast attenuation (CT value) of the esophageal wall following neoadjuvant chemoradiotherapy (nCRT) could improve diagnostic accuracy in the evaluation of the ESCC’s response to nCRT. The post-contrast attenuation value also predicted response more accurately than esophageal wall thickness. The esophagus wall may have continued to grow as a result of the nCRT, although the modest esophageal wall thickness and consistently low post-contrast attenuation may imply foci regression. The esophagus wall frequently remained thickened following nCRT. However, the moderately thickened wall and consistently modest post-contrast attenuation may imply foci retreat. According to Li’s study (30), the average post-contrast density of the esophageal wall in the area of the tumor that had previously been present after CRT was 64.35 ± 12.89 HU, with an average CT value of 23.86 ± 10.59 HU. Additionally, Yang Li’s report (31) found an association between the tumor’s CT value and the lymphatic vascular invasion status. Based on the studies above, a number of risk factors were included in the COX analysis. Univariable analysis of the training cohort revealed that the following factors were all prognostic predictors for LRFS: tumor site, history of alcohol usage, TL, TV, T stage, N stage, and GTV dose, whether chemotherapy was received, MWT before RT, MWT after RT, NS before RT, NS after RT, CT value before RT and ΔCT value. We eventually developed a nomogram for ESCC patients based on the outcomes of multivariate analysis that incorporates the T stage, N stage, GTV dose, tumor location, MWT after RT, NS after RT, ΔCT value, and whether patients received chemotherapy.

We assessed the nomogram’s capabilities for identification, calibration, and efficacy, and we contrasted them with the 8th AJCC TNM stage’s capacity for prediction. The C indices for the two nomogram groups were 0.705 and 0.699, respectively, while the C indices for the TNM were 0.572 and 0.601. On both the training and validation datasets, the forecasting model showed greater 1-, 3-, and 5-year AUCs for LRFS, which was superior to the staging criterion for the 8th TNM, demonstrating a good capacity for prediction. The C-index and AUC curve for the prediction model based on clinical characteristics significantly improved when with reference to the 8th TNM staging criterion on both the training dataset and validation dataset, suggesting that it may be better able to predict the LRFS in ESCC patients. Additionally, both on the training and validation datasets, the forecasting model demonstrated high calibration capabilities. In **Figure 4**, a clinical decision curve analysis was displayed. Both the training dataset and the validation dataset contain predictions that most patients can profit from. This was in line with what previous reports had discovered. Luo (15) combined radiomics signature Rad-score and clinical staging to provide an economical and simple method for predicting the CR status of CRT for patients with ESCC, and the nomogram model outperformed clinical staging significantly. To forecast the prognosis of individuals with ESCC who get CRT, Chen (25) built and verified a new model that incorporates the parameters of the tumor’s length, thickness, nutritional index, TNM stage, and inflammatory index. This model outperformed the eighth TNM staging standards, and it was suggested that prognostic variables for ESCC should consider the tumor thickness and the length tumor. As a result, the nomogram model we developed may be a better predictor of LRFS among ESCC patients who received radical (chemo) radiotherapy than just using the 8th TNM staging criteria. The nomogram is able to forecast the outcome of ESCC patients receiving RT and serve as a foundation for doctors to develop treatment plans.

Overall, the nomogram we developed in the current investigation was effective at predicting LRFS in patients with ESCC. Furthermore, patients with varying levels of risk could be distinguished with accuracy using the nomogram’s score. The risk classification technique based on the nomogram score performed well in terms of prediction. The anticipated LRFS nomogram allowed for the classification of patients enrolled into low, medium, and high-risk categories. Significant distinctions were seen between these three groups in terms of local recurrence, regional lymph node failure, and/or mortality. According to non-textbook outcomes, Xu (19) developed a nomogram for the prognosis of patients with ESCC after minimally invasive esophagectomy. This nomogram could accurately predict overall survival (OS) and disease-free survival (DFS) in ESCC patients after minimally invasive esophagectomy (MIE), and the nomogram score might give adequate risk classification for ESCC patients following MIE. These findings suggested that the nomogram-based risk stratification offered a trustworthy method for predicting prognosis, which might be a significant addition to the TNM staging approach. Importantly, the “observation and waiting” strategy might be applicable to low-risk patient groups. For ESCC patients assessed as high-risk, closely followed up, and individual, active treatment was particularly important for disease management.

There were certain restrictions on the current investigation. First, a single-center retrospective analysis served as the study’s foundation. As a result, the outcomes can be skewed, and an external verification from other centers or future data would enhance the generalizability and robustness of the findings. Second, the patients in our study only had ESCC; there is no general application to patients with other forms of EC. Third, compared to the worldwide esophageal cancer collaboration database, the size of this study was very small; therefore, further research using larger sample sizes is required to support our findings. Furthermore, the LRFS follow-up was partially decided by patients or family members, which could lead to biased analysis.

## 5 Conclusion

In conclusion, for ESCC patients who received radiotherapy, the nomogram based on clinical risk factors could reliably predict the LRFS, which demonstrated high predictive power and may be more effective than the 8th TNM staging criterion. Close follow-up and aggressive treatment are especially crucial for illness management patients with ESCC who were deemed to be at high risk.

## Data Availability

The raw data supporting the conclusions of this article will be made available by the authors, without undue reservation.
